# Whole-Cell Biosensor for Iron Monitoring as a Potential Tool for Safeguarding Biodiversity in Polar Marine Environments

**DOI:** 10.3390/md22070299

**Published:** 2024-06-28

**Authors:** Marzia Calvanese, Caterina D’Angelo, Maria Luisa Tutino, Concetta Lauro

**Affiliations:** 1 Department of Chemical Sciences, University of Naples “Federico II”, Complesso Universitario Monte S. Angelo, Via Cintia 4, 80126 Naples, Italy; marzia.calvanese@unina.it (M.C.); caterina.dangelo@unina.it (C.D.); tutino@unina.it (M.L.T.); 2 Istituto Nazionale Biostrutture e Biosistemi (I.N.B.B), Viale Medaglie D’Oro 305, 00136 Roma, Italy

**Keywords:** iron monitoring, polar marine environments, whole-cell biosensors, marine bacteria

## Abstract

Iron is a key micronutrient essential for various essential biological processes. As a consequence, alteration in iron concentration in seawater can deeply influence marine biodiversity. In polar marine environments, where environmental conditions are characterized by low temperatures, the role of iron becomes particularly significant. While iron limitation can negatively influence primary production and nutrient cycling, excessive iron concentrations can lead to harmful algal blooms and oxygen depletion. Furthermore, the growth of certain phytoplankton species can be increased in high-iron-content environments, resulting in altered balance in the marine food web and reduced biodiversity. Although many chemical/physical methods are established for inorganic iron quantification, the determination of the bio-available iron in seawater samples is more suitably carried out using marine microorganisms as biosensors. Despite existing challenges, whole-cell biosensors offer other advantages, such as real-time detection, cost-effectiveness, and ease of manipulation, making them promising tools for monitoring environmental iron levels in polar marine ecosystems. In this review, we discuss fundamental biosensor designs and assemblies, arranging host features, transcription factors, reporter proteins, and detection methods. The progress in the genetic manipulation of iron-responsive regulatory and reporter modules is also addressed to the optimization of the biosensor performance, focusing on the improvement of sensitivity and specificity.

## 1. Introduction

Covering more than 70% of the Earth’s surface, the oceans provide habitat for a variety of life forms, from microscopic plankton to majestic whales, each playing a unique role in maintaining the health and balance of marine ecosystems. The intricate interplay among these diverse organisms not only sustains the ocean food web but also contributes significantly to global ecological stability. Marine biodiversity provides invaluable ecosystem services, such as carbon sequestration, oxygen production, coastal protection, and nutrient cycling [1]. However, escalating human activities, including overfishing, habitat destruction, pollution, and climate change, pose significant threats to marine biodiversity, endangering not only marine species but also the intricate ecological processes that support life on our planet [2]. Thus, safeguarding marine biodiversity emerges as a pressing imperative for ensuring the long-term health and sustainability of marine ecosystems worldwide.

Iron (Fe) is one of the most abundant elements on Earth that plays a critical role in marine environments and biodiversity [3,4]. It exists in four different states: 0, +II, +III, and +VI. However, the reduced ferrous Fe^2+^ and oxidised ferric Fe^3+^ forms predominate. Due to its capability to adopt different spin states and exchange electrons, iron is an essential cofactor and prosthetic component in proteins involved in many major biological processes. These include photosynthesis, nitrogen fixation, methanogenesis, the production and consumption of H_2_, the respiratory electron transport chain, the tricarboxylic acid (TCA) cycle, gene regulation, and DNA biosynthesis [5,6,7,8,9,10,11]. The role of iron in such bio-geochemical processes explains why it is an essential micronutrient for many forms of marine life.

Unlike the terrestrial environment, iron concentration in the open ocean is extremely low, and its distribution in non-hydrothermal regions is uneven, ranging from 0.05 to 0.2 nmol L^−1^ [12,13,14,15]. This is because the soluble ferrous form tends to be rapidly oxidized to thermodynamically favoured but insoluble ferric species in seawater [16]. Moreover, this form frequently converts to oxyhydroxide complexes, which subsequently adsorb each other, precipitating as a particulate matter [13]. 

The consequences of iron scarcity in marine environments have been extensively studied, focusing on metabolic remodelling in organisms like phytoplankton [17], heterotrophic bacteria [18], diazotrophs [19], and diatoms [20,21,22]. In general, Fe-limitation conditions are associated with a reduction of the capability to produce ATP [23] and regulations in metabolic pathways, such as the TCA cycle, glycolysis, oxidative phosphorylation, glyoxylate cycle, and photosynthesis [18,24,25]. This limits the growth and development of photosynthetic organisms, which constitute the basis of the marine food chain. Reduced primary production can affect food availability for zooplankton, fishes, and superior organisms, thus influencing the structure and dynamics of the marine biological community [26,27,28,29]. Moreover, iron deficiency affects the carbon cycle by reducing the absorption of CO_2_ from the oceans, potentially contributing to global warming, ocean acidification, and the formation of dead zones [30,31]. This is typically observed in high-nutrient low-chlorophyll (HNLC) regions of the Southern Ocean. In these regions, iron fertilization experiments, where iron is deliberately added to iron-deficient waters, have been conducted, resulting in phytoplankton blooms and the stimulation of bacterial growth [32,33,34,35]. These experiments support the “Iron hypothesis”, which posits that in HNLC regions, the abundance of nutrients like nitrate and phosphate is high, but the scarcity of iron limits primary productivity [36].

Interestingly, the dissolved iron concentration is highly variable in marine waters, particularly the higher closer to land and/or surrounded by land masses than the open ocean. Furthermore, recent studies revealed that this variability is also affected by meltwater produced by global warming, supplying iron in a variable fashion within seawater, with the possible formation of iron-rich areas [37,38,39]. Along with the iron limitation, the iron excess can also induce toxicity in marine organisms. Toxicity experiments performed on mesocosms of benthic macroinvertebrates demonstrated a Final Chronic Value of ferric iron of 4.48 µM L^−1^ [40]. In general, the deleterious effects can be multiple: cellular membranes are damaged, compromising structural integrity and cellular functions, while metabolic processes, including respiration, photosynthesis, and the synthesis of essential biomolecules, are inhibited. Oxidative stress occurs as well, with the formation of highly reactive free radicals that damage DNA, proteins, and cellular lipids [10,41]. Extremely high concentrations of iron can lead to the mortality of marine organisms, resulting in mass mortality events [42,43]. 

Hence, both iron excess and deficiency can alter the composition and abundance of marine biological communities, favouring the growth of organisms capable of tolerating low or high iron concentrations at the expense of more sensitive species [44,45]. As a consequence, the dissolved iron concentrations in seawater can significantly impact the biodiversity of the ecosystem. The crucial role of iron in marine biodiversity is particularly emphasised in microbial communities living in the Southern Ocean. The Southern Ocean biological pump, which involves the uptake of inorganic carbon through photosynthesis and the export of organic carbon to the ocean depths, is mediated by both eukaryotes and prokaryotes. Specifically, planktonic photosynthesis is limited by the availability of light and essential trace metals such as iron, especially in HNLC areas. However, certain regions of the Southern Ocean can become seasonally productive, experiencing phytoplankton blooms dominated by diatoms, Phaeocystis, or nanoplankton, observed near Antarctic and sub-Antarctic islands and during the seasonal retreats of sea ice. Climate change is altering the availability of iron and the structure of marine ecosystems in the Southern Ocean, leading to longer ice-free periods, increased stratification of the upper oceans, and enhanced light availability. These factors could increase primary productivity and improve carbon export to the ocean depths, globally affecting nutrient availability and biological productivity [46].

Marine prokaryotes, including bacteria, archaea, and viruses, play fundamental roles in CO_2_ sequestration, nitrogen cycling, and supporting local and global food webs. The microbial carbon pump (MCP) is an important mechanism for long-term carbon storage in the deep oceans, with bacterial communities oxidizing recalcitrant dissolved organic matter. Iron availability influences microbial community dynamics and ecosystem functions, with rapid bloom responders like SAR92 and Aurantivirga dominating bacterial compositions in diatom blooms due to efficient iron uptake strategies [46].

These findings underscore the complexity and uniqueness of marine communities in the Southern Ocean, highlighting their adaptation to distinctive environmental conditions and their crucial roles in such an ecosystem. Monitoring iron availability in these environments is essential for understanding and predicting changes in marine biodiversity and the functioning of oceanic carbon cycles, particularly in the face of ongoing climate change.

This review describes the potentiality of the ongoing monitoring of iron levels in marine environments as a tool to understand and mitigate its impacts on marine biodiversity, focusing on the exploitation of bacterial biosensors.

## 2. Methods for Iron Monitoring in Seawater

Considering the reasons mentioned above, the determination of the concentration of dissolved iron is of great interest to the scientific community. Iron solubility depends on diverse factors such as temperature, pH, and salinity besides the oxidative state [13]. In addition, global warming and climate change strongly contribute to the iron level variations in seawater. Hence, attention has also shifted to iron fluxes from glaciers and sea ice to relevant local sources of iron with increasing melt caused by global warming [37,38,47]. Initiatives like the International GEOTRACES Program contribute significantly to unravelling the complexities of the oceanic iron cycle [48,49]. All these environmental variations make the determination of iron in oceans a challenging task [50,51].

The main techniques applied for iron monitoring in seawater are reported in Table 1. Lin et al. compared some of them in terms of complexity, sensitivity, and applicability [52]. Techniques like atomic absorption spectrometry (AAS) and inductively coupled plasma mass spectrometry (ICP-MS) are characterized by high sensitivity and can be particularly useful for detecting very low iron concentrations, even down to 14 pmol L^−1^ [53,54]. However, the typical iron concentrations in HNLC regions (0.03–0.07 nmol L^−1^) pose a challenge as they approach the lower detection limits of these techniques [34]. On the other hand, laboratory methods such as spectrophotometry [55,56] and voltammetry [57,58] offer cost-effective and straightforward analysis. While voltammetry is characterized by high sensitivity up to 5 pmol L^−1^ [51], the spectrophotometric methods, with detection limits often higher than those required for HNLC regions, might need pre-concentration steps to be applicable in such low-iron environments. Indeed, these methods rely on the formation of coloured complexes by iron and specific ligands, as well as the electrical current associated with iron reduction or complexation, respectively. Moreover, chemiluminescence, based on light emitted during the reaction of iron with specific chemicals, is routinely used to measure dissolved iron in seawater with a good level of sensitivity (40 pmol L^−1^) [59,60], making it more suitable for HNLC conditions.

Onboard ships, the direct detection of iron levels is also feasible. These methods prioritize simplicity, automation, and sensitivity, aiming to minimize sample preparation and storage requirements. They facilitate efficient and continuous analysis, particularly for low iron concentrations, ensuring accurate results even in challenging marine environments. Flow Injection Analysis (FIA) stands out as a popular technique due to its ease of automation and high sample throughput [61]. It involves the injection of the sample into a flowing carrier stream that gives rise to a transient signal at the detector, which is dependent on the physical and chemical kinetic processes occurring inside. Combined with chemiluminescence, FIA enables precise measurements in acidified seawater using a lab-on-valve (LOV) format, which is a compact multi-purpose flow cell located atop a multi-position valve used for the miniaturization of FIA systems [62]. Recent advancements, including Long Path Length Liquid Waveguide Capillary Cell (LWCC) and Reverse Flow Injection Analysis (rFIA), have further improved detection limits, enhancing the reliability of iron speciation analysis up to 0.1–0.4 nmol L^−1^ [63,64]. LWCC is a technique that harnesses a flow cell for absorbance measurements in the ultraviolet and visible ranges of small-volume samples, while rFIA is an FIA mode applied to multi-component analysis by the sequential injection of different detection reagents into a mobile phase of the sample. These improvements make FIA techniques potentially suitable for analysing iron in HNLC regions, although the lower end of HNLC iron concentrations may still be challenging. Looking ahead, voltammetry coupled with FIA and innovations in 3D printing enable the cost-effective fabrication of automated systems, signalling a bright future for iron speciation analysis in marine environments [65,66].

In the field of the direct measurement analysis of iron speciation in seawater, in situ methods are increasingly gaining interest for long-term iron monitoring, which is crucial for advancing the understanding of the spatial and temporal distribution of iron speciation in seawater. Traditional techniques like ICP-MS and AAS, although accurate, are hindered by their lack of portability and complex sample preparation requirements. Similarly, colourimetric and chemiluminescence methods, while offering speciation capabilities and lower detection limits, require pre-concentration and matrix removal, making them less suitable for in situ analysis. Emerging technologies like the Voltammetric In Situ Profiling System (VIP) and the Multi Physical Chemical Profiler (MPCP) enable the continuous in situ analysis and speciation of iron based on redox state and binding properties. Both techniques rely on immersible probes containing a mini-voltammetric cell able to measure the electrical current associated with various iron species: the VIP system consists of an array of 100 interconnected Ir-based micro-disc electrodes known as Gel-Integrated MicroElectrode (GIME sensor), while MPCP couples the GIME sensor to a submersible FIA system [67]. The main advantage of VIP and MPCP is the capability to overcome sensor reliability, underwater pressure, and fouling issues, allowing for reliable monitoring for up to 8 days. In addition, biosensors based on fluorescence-quenching using siderophore for Fe(III) detection [68], infrared spectral changes of immobilized iron-chelating desferrioxamine B (DFB) upon Fe(III) complexation [69], and autonomous spectrophotometric determination of the red-purple complex formed by Fe(II) and ferrozine [70] provide alternative and simple methods for the real-time monitoring of iron in the marine environment. However, all these methods suffer from instability in long-term monitoring related to chip deterioration and biofouling and require further optimization for concrete field applications.

The chemical speciation of iron can change rapidly once the water is sampled and separated from its natural environment. Hence, the increasing need to rapidly measure iron in environmental waters has led to the development of real-time monitoring techniques. One such method combines a novel electronic tongue integrated with multiple light-addressable potentiometric sensors (MLAPS) and stripping voltammetry [71]. This approach capitalizes on the high sensitivity of stripping voltammetry and the spatial resolution of MLAPS. Stripping voltammetry, including anodic stripping voltammetry (ASV) and adsorptive cathodic stripping voltammetry (CSV), can measure trace metals with minimal sample requirements but often faces challenges like peak overlaps and intermetallic compound formation. The MLAPS addresses these issues by using light-addressability to influence only a limited area of the sensitive film, enabling accurate multi-metal detection. Additionally, advancements in ion-selective chalcogenide glass sensors enhance the chemical stability and selectivity of MLAPS, particularly for heavy metals like Fe (III). Another example is the portable flow injection chemiluminescence (FI-CL) instrument designed by Bowie and colleagues for the online monitoring of iron (II) in surface seawater [72]. The instrument leverages the catalytic effect of iron (II) on the luminol reaction without the need for added oxidant. It can also measure dissolved iron (II + III) with minor modifications to the manifold and software, and it was successfully applied for shipboard trials conducted over several day–night cycles during a north–south transect in the subtropical Atlantic Ocean and a daytime transect in the Sub-Antarctic Front south of Australia. Furthermore, the development of the “IonConExplorer” allowed for the real-time in situ analysis of deep-sea iron detection using long pathlength absorbance spectroscopy (LPAS) coupled with a liquid waveguide capillary cell (LWCC) [73]. This instrument provides high sensitivity and low detection limits suitable for nanomolar concentrations of iron, which is crucial for understanding ocean biogeochemistry, including HNLC regions. Moreover, a novel automated smart sensor powered by solar energy has been recently described [74]. It employs potentiometric Fe^2+^-selective electrodes, offering low-cost, long-term monitoring capabilities with rapid response times and significant resistance to interference [74]. It shows high sensitivity (29.76 ± 0.6 mV per decade), a wide concentration range (10^−1^–10^−5^ mol L^−1^), and rapid response (<3 s) with high selectivity among various metal ions. The autonomous power source and GPS navigation ensure continuous operation and precise location control.

However, analytical techniques often fail to provide specific information regarding bioavailable iron. The term ‘bioavailable’ refers to the fraction of iron that is accessible and usable by marine organisms for biological processes. This is in contrast to total iron, which includes both bioavailable and non-bioavailable forms. As a consequence, understanding the dynamics of bioavailable iron in marine ecosystems can aid in elucidating microbial processes and ecosystem functioning. In this context, whole-cell biosensors (WCBs) constitute a promising class of real-time biosensors based on bacteria genetically engineered to respond specifically to different iron species. Such technology represents an alternative for more simple and cost-effective in situ iron analysis [75]. The advantages of WCBs are numerous, and first and foremost is the high specificity. WCBs, indeed, can be designed to target specifically bioavailable iron species with minimal interference from other metals or compounds present in seawater. This concept of biosensor was pioneered by Selifonova et al., who demonstrated the use of genetically engineered bacteria to detect bioavailable forms of mercury in environmental samples [76]. Furthermore, WCB can be miniaturized and made portable, potentially enabling in situ monitoring in remote or challenging environments where traditional sampling methods may be impractical [77,78,79].

**Table 1:** Methods for iron monitoring in seawater.

Method	Measurement	Benefits	Drawbacks	Detection Limit
Atomic Absorption Spectrometry (AAS)	Laboratory	High sensitivity Short detection time	Pre-concentration of samples High sample volume Cumbersome equipment Expensive costs	50 pmol L^−1^ [80]
Inductively Coupled Plasma Mass Spectrometry (ICP-MS)	Laboratory	High sensitivity Short detection time Small sample volume	Pre-concentration of samples Cumbersome equipment Expensive costs	14 pmol L^−1^ [54]
Spectrophotometry	Laboratory	Iron speciation selectivity Simple procedure and data analysis Short detection time Inexpensive	Limited sensitivity Interference by coloured contaminants Requirement of stable iron complexes Pre-treatment of samples	1.9 nmol L^−1^ [81]
Voltammetry	Laboratory	High sensitivity Fast and simple procedure Iron speciation selectivity	Interference by other heavy metals Pre-treatment of samples Expensive maintenance costs	5 pmol L^−1^ [51]
Chemiluminescence	Laboratory	High sensitivity Iron speciation selectivity Short detection time Wide dynamic range Inexpensive	Interference by other chemical species Pre-treatment of samples Matrix removal requirement	40 pmol L^−1^ [60]
Flow Injection Analysis (FIA)	Onboard ship	High sensitivity Easy automatic operation Short detection time High sample throughput Low reagent consumption Minimizes the redox change and contamination	Expensive instrumentation Pre-treatment of samples Matrix removal requirement	25 pmol L^−1^ [82]
Long Path Length Liquid Waveguide Capillary Cell (LWCC)	Onboard ship	High sensitivity Easy automatic operation High sample throughput Iron speciation selectivity Small sample volume Background signal reduction	Expensive costs Sensitivity to impurities Pre-treatment of samples	0.1 nmol L^−1^ [63]
Reverse Flow Injection Analysis (rFIA)	Onboard ship	High sensitivity Easy automatic operation High sample throughput Fast and precise measurements Low reagents consumption Suitable for long-term shipboard use	Expensive instrumentation and maintenance costs Pre-treatment of samples Matrix removal requirement	0.4 nmol L^−1^ [64]
Voltammetric In Situ Profiling System (VIP)	In situ	Iron speciation selectivity Immersible in seawater Minimizes sample volume High spatial and temporal resolution	Expensive costs Long-term instability Low data accuracy for long-term operation	0.27 nmol L^−1^ [67]
Multi Physical Chemical Profiler (MPCP)	In situ	Iron speciation selectivity Immersible in seawater High spatial and temporal resolution Multiparameter measurements Easy automatic operation Minimize sample volume	Expensive costs Long-term instability Low data accuracy for long-term operation	0.2 nmol L^−1^ [67]
Whole-Cell Biosensor (WCB)	In situ	Bioavailable iron measurement High sensitivity Simple manipulation Inexpensive Potentially suitable for real-time measurements	Long-term maintenance Environmental containment Environmental interference Limited resolution Limited response time	40 pmol L^−1^ [34]
Multiple Light—Addressable Potentiometric Sensors (MLAPS)	In situ real-time	High sensitivity when coupled with voltammetry High specificity Fast detection speed Easy automatic operation Minimal sample requirement Multianalyte measurements	Expensive costs Limited measurement accuracy in complex environments Interference by multiple heavy metals Long-term stability	50 nmol L^−1^ [71]
Long Pathlength Absorbance Spectroscopy (LPAS)	In situ real-time	High sensitivity when coupled with LWCC Precision and Accuracy Minimal sample requirement Minimal interferences Easy automatic operation Suitable for deep sea monitoring	Expensive costs Long-term stability Frequent system maintenance	27.25 nmol L^−1^ [73]

## 3. Whole-Cell Biosensors: Main Features and Key Elements

**Figure 1:**
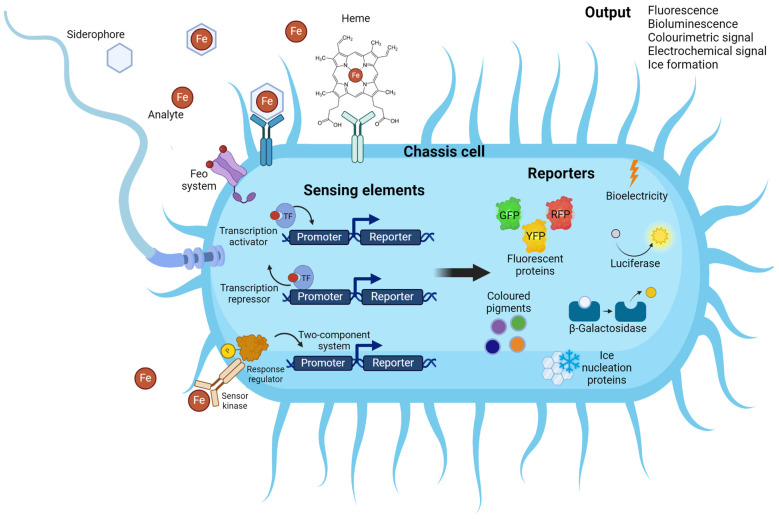
Whole-cell biosensor for iron monitoring. Iron is internalized within cells through mechanisms mediated by the siderophores uptake system, heme uptake system, or Feo system. Once inside, iron is sensed by transcription factors, including transcription activators (one-component or two-component regulators) and repressors, which regulate the expression of the reporter gene. Based on the applied reporter, a fluorescent, bioluminescent, colourimetric, or bioelectric output signal is obtained, allowing for the measurement of bioavailable iron. Created with graphical elements available on https://app.biorender.com/ (accessed on 8 April 2024).

Many marine organisms have evolved sophisticated mechanisms to regulate iron levels within their cells, often employing specific regulatory elements encoded by heavy metal resistance operons. These operons are activated in response to the presence of iron in the surrounding environment, triggering the expression of genes involved in iron uptake and homeostasis. By harnessing these natural regulatory components, an innovative biosensing technology has been developed. One such approach involves coupling these elements with suitable readout systems to construct WCBs. In these biosensors, the regulatory elements function as receptors, selectively capturing iron ions from the external environment. The captured ions then trigger the expression of a reporter gene, which generates a measurable output detected by the readout system (Figure 1) [83].

In general, the design of WCBs encompasses several key steps. The first one is the identification of metal sensing and response elements within the genomes of microorganisms capable of thriving in metal-rich environments. These elements often include transcription factors (TFs) capable of detecting specific target ions, thereby initiating cellular responses to changes in metal concentrations. Specifically, TFs bind promoters that drive the expression of effector proteins (EPs) aimed at mitigating metal toxicity. The second step in constructing a WCB is the integration of these elements into engineered bacterial strains, replacing EPs with reporter proteins. Once genetic circuits have been assembled, the engineered strains undergo environmental testing to assess their performance and reliability in real-world scenarios. Through iterative cycles of testing and refinement, researchers optimize the biosensor’s performance to achieve a robust and sensitive detection of target metals.

In addition to TFs, two-component regulatory systems (TCRS) have recently been introduced as biosensors [84,85]. While in one-component systems (TFs), the allosteric change occurs after direct detection of the target molecule, TCRS are characterized by a more complex response mechanism [86]. At the heart of the TCRS are the histidine kinases (HK), which are typically transmembrane proteins capable of detecting specific molecules and acting as sensor proteins. Upon sensing a stimulus, the kinase undergoes a conformational change, enabling its autophosphorylation. Subsequently, it transfers the phosphate group to a cytoplasmic response receptor (RR), which acts as a regulator protein. The activated regulator can then bind to its promoter, ultimately driving the expression of the reporter gene. Despite their versatility and the possibility of combining independent modules to create effective signalling circuits, the complexity of such systems is the main limitation to their concrete exploitation in WCB technology [87].

Other sensing elements feasible for WCB include riboswitches. Riboswitches can up- or down-regulate gene expression after binding to a specific, inducing a conformational change that either favours or hampers ribosome binding to the mRNA of the downstream controlled gene [88]. Since these regulatory elements act at a translational level, riboswitch-based WCBs are characterized by a faster response in comparison to TF and TCS, which require both transcription and translation. However, despite being well characterized, the application of riboswitches in whole-cell-based biosensors has been relatively limited [89,90,91].

### 3.1. Heavy Metal Sensor Elements

Most of the WCBs developed to detect heavy metals, including iron, use TFs as sensor elements (Table 2) [92,93]. Regulating the detoxification and homeostasis of heavy metals within bacterial cells, TFs can be primarily classified as activators and repressors, depending on their mode of action. After the effector metal detection, activators bind to promoters to induce conformational changes that allow RNA polymerase to initiate the transcription of downstream genes, while repressors inhibit translation through steric hindrance. The genes controlled by these TFs typically encode proteins involved in metal detoxification, as well as efflux or import pumps [94].

**Table 2:** Transcription factors used as sensor elements in WCBs for heavy metal detection.

Transcription Factor (TF)	Type	Genes Controlled	Organisms	WCB Applications	References
MerR	Activator	merA (mercury reductase), merB (permease), merC (metallothionein)	*E. coli*, *P. aeruginosa* PAO1, *P. putida*	Detection of mercury in seawater and lakes using GFP, RFP, violacein, pyocyanin, Luciferase	[95,96,97,98,99,100,101,102]
ArsR/SmtB	Repressor	arsA, arsB, arsC (E. coli); arsC, arsD, arsR (B. subtilis)	*P. aeruginosa* PAO1, *P. putida*, Enterobacteria, *E. coli*	Detection of arsenic in lakes and groundwater using GFP and β-Galactosidase; detection of cadmium, lead, antimony	[103,104,105,106,107,108,109,110,111,112,113,114,115]
Fur	Repressor/Activator	Iron-responsive genes, siderophore synthesis genes, ROS defence, respiration, chemotaxis, nitrogen metabolism, photosynthesis, virulence, glycolysis, citric acid cycle genes	*E. coli*, *Corynebacterium*, Streptomyces, *Mycobacterium*	Detection of iron in freshwater and South West Pacific using bioluminescence in *P. putida*	[34,116,117,118,119,120,121,122,123]
DtxR	Repressor/Activator	Iron uptake and metabolism genes	*Corynebacterium*, Streptomyces, *Mycobacterium*	N.D.	[122,123]
IscR	Repressor	iscSUA-hscBA-fdx iron–sulfur cluster	*E. coli*	N.D.	[124,125,126,127,128]

Among the activator TFs, MerR is a well-characterized metalloregulatory protein involved in bacterial resistance to mercury [95,96]. The activation mechanism of MerR involves several steps. Firstly, it binds to mercury at a designated site within its C-terminal domain. This binding induces a conformational change in the MerR protein, exposing its activation domain. This activated form interacts with RNA polymerase, enhancing its transcriptional activity of genes associated with mercury resistance, such as *merA*, *merB*, and *merC*. The former *merA* encodes a mercury reductase that converts ionic mercury (Hg^2+^) into elemental non-toxic mercury (Hg^0^), which can be easily expelled from the cell. On the other hand, *merB* encodes a permease, facilitating the excretion of elemental mercury, while *merC* encodes metallothionein that binds and sequesters mercury within the cell. The MerR regulator has been successfully utilized in designing WCBs for Hg monitoring in seawater and lakes. The versatility of this system has been extensively explored, leading to the development of numerous biosensors employing hosts such as *E. coli*, *Pseudomonas aeruginosa* PAO1, and *Pseudomonas putida* to express reporter proteins like GFP, RFP, violacein, pyocyanin, and Luciferase. These biosensors exhibit a good level of linearity in detecting Hg at nanomolar scales [97,98,99,100,101,102].

The most studied repressor TF family is ArsR/SmtB, which plays a crucial role in maintaining cellular defence mechanisms against various toxic metals, primarily arsenic and cadmium [103,104,105]. Under normal conditions, it binds to the promoter’s operator sequence of the *ars* operon, preventing the expression of genes responsible for dealing with heavy metals. However, when exposed to elevated levels of arsenic or cadmium, ArsR/SmtB acts as a metal sensor, detecting the threat and initiating a response. Initially, it exists as a dimer, with two identical protein subunits coming together. Specific regions, alpha3N or alpha5 sites, serve as binding pockets for metal ions. Upon metal binding, the protein undergoes a conformational change, leading to the detachment from the DNA it was previously repressing. This event allows for the activation of the *ars* operon genes, facilitating the production of proteins crucial for heavy metal resistance. Notably, the specific genes within the *ars* operon’s binding site may vary among bacterial species [106,107]. For instance, in *E. coli*, the *ars* operon encompasses *arsA*, *arsB*, and *arsC* genes, which encode proteins responsible for arsenic efflux from the cell. Conversely, in *B. subtilis*, the *ars* operon includes *arsC*, *arsD*, and *arsR* genes, involved in arsenic sequestration and the regulation of metal response pathways. Overall, the *ars* operon plays a fundamental role in bacterial adaptation to environments with elevated heavy metal concentrations, ensuring cellular survival and fitness. ArsR-based biosensors have been developed in different bacterial species such as *P. aeruginosa*, *P. putida*, and various Enterobacteria and *E. coli* strains [108,109,110]. Other WCBs have been utilized for the detection of bioavailable arsenic in samples sourced from lakes and groundwaters. Utilizing GFP and β-Galactosidase as reporters, these biosensors have demonstrated detection limits ranging from 100 to 1.85 nmol L^−1^ [111,112]. Moreover, the ArsR regulator has found applications in WCBs for detecting other heavy metals such as cadmium, lead, and antimony, further highlighting their utility in assessing water pollution and its effective management [113,114,115].

**Figure 2:**
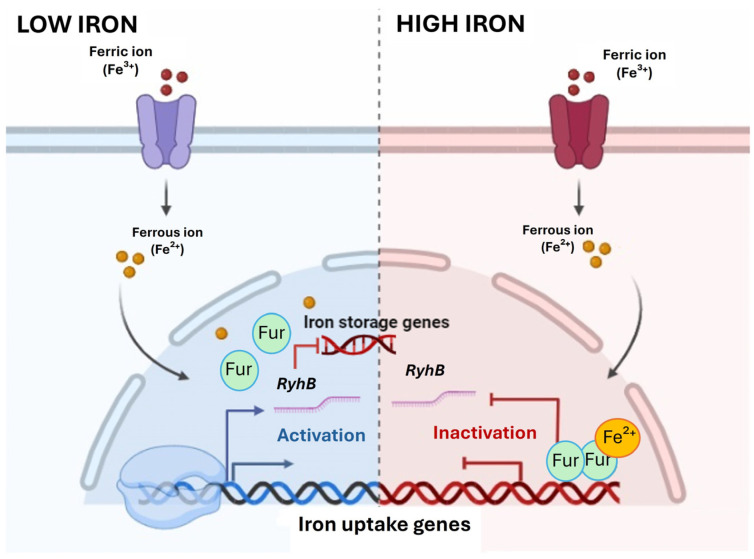
Iron uptake and metabolism regulated by Fur. In high iron conditions, ferrous iron (Fe^2+^) acts as a co-repressor, enhancing Fur DNA binding affinity and repressing genes involved in iron uptake to prevent toxicity. Fur can also activate genes expressing iron storage proteins by repressing the small non-coding RNA RyhB activity. Under low-iron conditions, Fur releases Fe^2+^ and dissociates from DNA, allowing the expression of siderophore synthesis genes. Without any interaction of Fur with RyhB, iron storage genes are repressed.

As for iron, its metabolism is mainly regulated by Fur (ferric-uptake regulator) in many bacteria, including marine species [116,117,118]. Fur controls the expression of numerous genes in an iron-dependent manner, modulating its intracellular usage (Figure 2). It is a dimer whose C-terminal domain is implicated in dimerization, while the N-terminus binds specific DNA sequences called Fur boxes, which are located upstream of promoters of iron-responsive genes. When the intracellular concentration of iron is plentiful, the ferrous iron (Fe^2+^) acts as a co-repressor, binding the Fur C-terminal domain and strongly increasing its affinity for DNA. This tight interaction results in the transcriptional repression of genes involved in active iron uptake, thus preventing potential toxic effects. Furthermore, Fur can also function as an activator by three main mechanisms: (1) interacting with small non-coding RNA (RyhB), (2) enhancing the recruitment of the RNA polymerase, and (3) acting as an antirepressor element, removing the transcription repression of some genes. These regulations result in the activation of genes participating in iron storage processes and Fe-binding enzymes [119,120]. Conversely, under iron scarcity conditions, Fur realises Fe^2+^ and dissociates from DNA, allowing the expression of genes required for siderophore synthesis, which are molecules that scavenge iron from the environment. Due to the critical role of iron in living organisms, the regulation of iron homeostasis mediated by Fur extends to the modulation of numerous other processes [121]. These include defence against damage caused by reactive oxygen species (ROS), pathways regulating essential processes such as respiration, chemotaxis, nitrogen metabolism, photosynthesis, virulence factor production, glycolysis, and the citric acid cycle. To date, over 90 regulated genes have been identified in association with these processes.

In some Gram-positive bacteria, such as *Corynebacterium*, *Streptomyces*, and *Mycobacterium*, DtxR (diphtheria toxin regulator) assumes the role of the primary iron regulator, stepping in for Fur [122,123]. DtxR and Fur exhibit similar functioning, binding to specific DNA sequences and controlling genes involved in iron uptake and metabolism. However, notable structural differences exist between the two proteins, mainly linked to the N-terminal DNA-binding domain that includes iron-sensing elements.

Another mechanism of intracellular iron management is the complexation of iron with sulfur, which is controlled by the Isc system in many bacteria [124,125,126]. In *E. coli*, this system involves genes encoded by the *iscSUA-hscBA-fdx* cluster, utilizing iron and L-cysteine as essential building blocks [127,128]. The regulation of this process is tightly controlled, with the IscR protein playing a pivotal role. IscR, containing its iron–sulfur cluster, modulates the expression of *iscSUA* genes through a negative feedback loop, adjusting cluster production to meet cellular demands. Intriguingly, this regulatory mechanism operates independently of the major iron regulator, Fur. Additionally, an alternative pathway, the *suf* operon, offers an alternative route for cluster formation under specific conditions regulated by Fur and activated during iron limitation or oxidative stress. These insights underscore the sophistication of biological iron–sulfur cluster assembly, with the Isc system serving as the primary pathway in *E. coli*, tightly regulated by IscR, while the Suf system offers an alternative route for adaptation to changing environmental conditions.

Intuitively, by manipulating genes under the control of these iron-dependent regulators, it is possible to design simple and reliable TF-based WCB for iron monitoring, as discussed in the examples described in Section 4.

### 3.2. Iron Internalization Mechanisms

**Figure 3:**
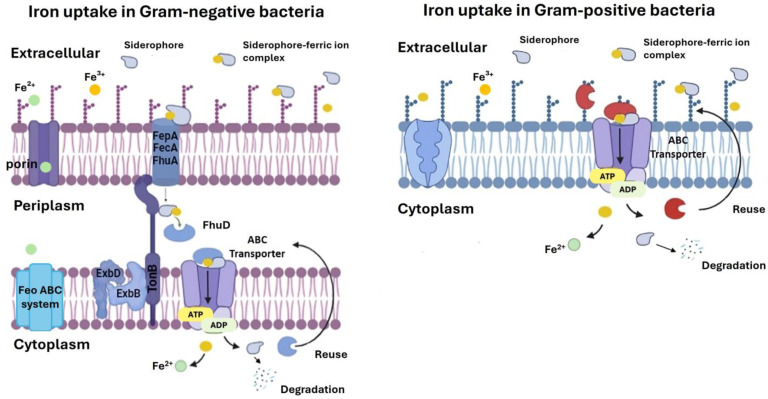
Iron uptake mechanisms in Gram-negative and Gram-positive bacteria. The Feo system is the main ferrous iron uptake mechanism used by Gram-negative bacteria, including FeoA, FeoB, and FeoC proteins. Most marine bacteria use siderophores for ferric iron uptake. Siderophores, secreted into the environment after their synthesis, form Fe^3+^ complexes, which are internalized through specific receptors. In Gram-negative bacteria, siderophore-ferric ion complexes are transported via outer membrane receptors (e.g., FepA, FecA, FhuA), interacting with the TonB-ExbB-ExbD complex for energy-dependent uptake and periplasmic binding proteins like FhuD. Gram-positive bacteria, lacking outer membranes, use simpler mechanisms involving extracellular membrane-anchored siderophore-binding proteins and ABC permeases. Once inside the cell, ferric iron is reduced to ferrous iron, while siderophores and membrane receptors can be reused or degraded. Created with graphical elements available on BioRender.com.

Holding mechanisms of iron uptake is pivotal for harnessing bacterial cells as efficient detection tools. To enable uptake even at low concentrations, specific internalization mechanisms characterized by high affinity must be activated. In marine waters, bacteria employ diverse strategies to internalize iron from their surroundings (Figure 3) [10,129,130,131]. These mechanisms are finely tuned according to the availability and chemical speciation of iron in the environment.

Ferrous iron is more abundant under anaerobic conditions or at low pH. A fundamental system for ferrous iron utilization is the Feo system, typically found in bacteria growing in low-oxygen environments [132,133,134]. Key components of this system in *E. coli* include FeoA, a cytoplasmic protein with an SH3-like domain; FeoB, a membrane protein consisting of a guanine nucleotide-binding domain at the N-terminus and an ATP/GTPase membrane domain in the C-terminus actively transporting iron; and finally FeoC, a small [Fe-S]-dependent translational receptor [132]. Despite FeoC being poorly conserved and lost in some bacteria species, the Feo system represents a major mechanism of iron internalization in several marine bacteria, including *Shewanella oneidensis* and *Vibrio cholerae* [135,136,137].

In environments where ferric ions predominate, marine bacteria mainly apply a strategy involving siderophores, small molecules with a high affinity for iron [129,130,131,138]. Siderophores are synthesized through either the nonribosomal peptide synthetase (NRPS) or the NRPS-independent synthetase pathways. NRPS are large, multi-enzyme complexes composed of modules, each responsible for incorporating a specific amino acid into the growing peptide chain. This modular structure allows for the incorporation of unusual amino acids and modifications, resulting in diverse and complex peptide products [139]. In contrast, NRPS-independent synthetases, also known as NRPS-independent siderophore synthetases (NIS synthetases), are enzymes that use a carboxylic acid substrate, typically citrate, or a derivative, and then catalyse the nucleophilic capture of an amine or alcohol [140]. After the synthesis, siderophores are secreted into the environment, where they form Fe^3+^ complexes that are subsequently re-internalized through specific receptors [140,141]. The specificity and affinity of interaction are so high to enable cells to acquire iron even under low-concentration conditions. Based on their structural features, two main types of marine siderophores are prevalent: amphiphilic and α-hydroxy carboxylic acid [129,142]. Amphiphilic siderophores are more abundant in surface seawater and are constituted by a hydrophilic head group associated with fatty acids. While the amino acid-based head group is highly conserved and is responsible for the iron chelation, the fatty acid length and degree of unsaturation or hydroxylation differ in each specific siderophore. On the other hand, α-hydroxy carboxylic acid siderophores, featuring an α-hydroxy carboxylic acid moiety such as β-hydroxy aspartic acid or citric acid, exhibit photoreactive properties when coordinated with Fe^3+^ and are commonly found in the photic zone [143]. Examples of amphiphilic types are Marinobactins, Imaqobactin, and Halochelins [144,145,146], while known α-hydroxy carboxylic acid siderophores include Aerobactin, Petrobactins, Aquachelins, and Woodybactin [147,148,149,150].

Once complexed with iron, siderophores undergo internalization through specific membrane receptors. This process differs between Gram-negative and Gram-positive bacteria [129]. In Gram-negative bacteria, these receptors are integral membrane proteins that recognize specific ferric–siderophore complexes at the cell surface [10,151,152]. Siderophore-ferric ion complexes are actively transported across cell membranes through an energy-dependent system involving outer membrane siderophore receptors, periplasmic binding proteins, and inner membrane transporters. Known outer membrane receptors are FepA, FecA, and FhuA, isolated and characterized from *E. coli* [153,154,155]. Structurally, they consist of two domains: a C-terminus comprising an integral anti-parallel β-barrel forming large extracellular loops and an N-terminus filling the interior of the barrel. This N-terminal domain serves as a plug and includes a region involved in siderophore binding. Intuitively, such receptors differ in this region since it is specific for siderophore recognition. Upon binding of the ferric siderophore, the outer membrane receptor interacts with the TonB-ExbB-ExbD complex, which is crucial for supplying the energy required to induce a conformational change of the receptor and the transport across the outer membrane [152,156,157]. In *E. coli*, this interaction is mediated by a conserved hydrophobic segment known as the TonB box located at the N-terminus of the outer membrane receptor. TonB is a periplasmic protein anchored to the cytoplasmic membrane by its hydrophobic N-terminal domain working in conjunction with the integral membrane proteins ExbB and ExbD. It is suggested that ExbB and ExbD use the membrane electrochemical charge to energize TonB. This state induces a conformational change of the outer membrane receptor bound to the siderophore. This change helps move the ferric–siderophore into the periplasm. Afterwards, TonB returns in a de-energized state and is recycled for further rounds of transport. After the ferric–siderophore complex has been released into the periplasm, high-affinity periplasmic binding proteins, like FhuD from *E. coli*, facilitate the transport to the cytoplasmic membrane [158]. The transport process occurs through a shallow pocket located between two lobes of the protein, which accommodates the ligand by the interaction between the iron–hydroxamate centre and residues within the binding pocket. Unlike classical periplasmic binding proteins involved in sugar and amino acid transport, FhuD lacks a flexible hinge region and does not undergo significant conformational changes upon ligand binding. Instead, the interaction relies on the iron-binding functional group, allowing FhuD to interact with diverse siderophores. Subsequently, a cytoplasmic membrane transporter belonging to the ATP-binding cassette (ABC) protein family enables the transport of iron–siderophore into the cytoplasm [159]. This process is driven by ATP hydrolysis that induces conformational changes in two transmembrane domains, creating a channel through which the Siderophore-ferric ion complexes are transported. Once the complex has been internalized, a class of enzymes known as ferric reductases catalyses the reduction of ferric ions to ferrous form, which exhibits a lower affinity for siderophores and dissociates from them [160]. One notable example of a ferric reductase commonly found in bacteria is ferredoxin, which plays a crucial role in mediating the reduction of ferric iron to its ferrous form [161,162].

In contrast, Gram-positive bacteria lack outer membranes and, consequently, the TonB-ExbB-ExbD complex. Therefore, they utilize a simpler mechanism for iron incorporation, where siderophores bind iron from the environment, and extracellular membrane-anchored siderophore-binding proteins associated with ABC permeases facilitate their transport into the cytoplasm [163]. Within cells, iron is released from the complex through reduction, similar to Gram-negative bacteria.

Interestingly, some marine bacteria can internalize exogenous siderophores produced by other organisms, further expanding their iron acquisition repertoire [164]. These “cheater” bacteria gain an advantage in terms of growth and fitness without incurring the costs of siderophore production [165,166].

Furthermore, some bacteria can use heme proteins in seawater as a direct source of iron. Within marine bacterial communities, two primary heme uptake systems have been identified: direct uptake systems and hemophore-mediated uptake systems [167]. Direct uptake systems employ specialized transporters to specifically facilitate the entry of heme into bacterial cells, while the second mechanism is very similar to siderophores’ energy-dependent uptake [168,169,170]. Once internalized, heme undergoes an oxidation process catalysed by heme oxygenases, allowing iron release from the porphyrin ring structure [171].

While the regulation of iron absorption is meticulously controlled based on intracellular iron needs, with the Fur protein playing a key role in siderophore-dependent uptake, as discussed above, nutrient availability seems to have minimal impact on siderophore production [172]. However, the production of siderophores is intricately linked to environmental conditions. Ocean acidification, a consequence of rising CO_2_ levels, poses a significant concern for iron bioavailability in marine ecosystems. While lower pH levels can enhance iron solubility, the presence of free hydroxide ions competes with siderophores for iron binding, potentially hampering siderophore production and bacterial growth. A recent study delved into the effects of iron concentration, temperature, and pH on siderophore production by marine bacteria from the Southern Ocean, demonstrating that lower pH levels had a detrimental impact on both growth and siderophore production [172]. Another regulatory mechanism based on quorum sensing plays a role in repressing the production of amphiphilic siderophores, as observed in the marine bacterium *V. harveyi* [173].

These mechanisms collectively demonstrate the adaptability of marine bacteria to scavenge iron efficiently, ensuring their viability in iron-limited marine ecosystems and their potential as a source of genetic elements to design efficient WCBs.

### 3.3. Reporter Genes

In a WCB, the iron-responsive promoter/operator controls the expression of a reporter, which generates a measurable signal in response to the analyte detection. The careful selection and utilization of suitable reporting elements are crucial for achieving accurate and reliable detection in a WCB (Table 3). Among the most widely employed reporters are those associated with optical readout, facilitating the rapid and straightforward measurement of the sensing response.

Bacterial luciferase (Lux), firefly luciferase (Luc), and aequorin are the main bioluminescence-based reporter proteins. Lux, encoded by the *luxA* and *luxB* genes within the *lux* operon, relies on the *luxCDE* genes to produce a long-chain aldehyde used as its substrate. The oxidation of this substrate results in the emission of a blue-green light measurable at 490 nm [174,175]. The ability of the luxABCDE system to generate luminescence signals without the addition of exogenous substrates makes it an optimal reporter for WCB. With properties similar to Lux, Luc catalyses the production of visible light in the presence of ATP, oxygen, and magnesium ions using luciferin as the substrate, whereas aequorin, derived from bioluminescent *Victoria* jellyfish, emits blue light upon the oxidation of coelenterazine in the presence of calcium ions [176,177]. The advantage of these reporters stands in their high sensitivity, signal stability, and absence of endogenous expression. However, it should be noted that Lux tends to be heat-labile, whereas firefly luciferase requires external substrates.

Fluorescent proteins represent another widely used reporting element, owing to their stability and ease of expression by a single gene [178,179]. Furthermore, the measurement of fluorescence can be easily performed by a fluorometer using specific excitation wavelengths. Among fluorescent proteins, the GFP has found extensive application in WCBs, primarily because it requires only adequate oxygenation for its maturation, making it highly compatible with bacterial cell metabolism [178]. To overcome its limitations, such as the extended time needed for fluorophore formation and high background signal, GFP was extensively studied and optimized, generating variants with higher sensitivity, brightness, and protein maturation rates [178]. However, the effective exploitation of GFP and its optimized variants in WCBs necessitates careful consideration of various factors beyond their inherent fluorescence properties. Key concerns include their compatibility with bacterial cell metabolism, photostability during detection processes, non-toxicity to host cells, and resilience to environmental fluctuations within the detection system [180]. Meeting these requirements, the utilization of GFP-based WCBs remains prevalent, driven by their proven efficacy in detecting bioavailable species and their adaptability to diverse recombinant genetic contexts. Furthermore, the availability of proteins with diverse fluorescent properties, such as red fluorescent protein (RFP), cyan fluorescent protein (CFP), and yellow fluorescent protein (YFP), enables the simultaneous detection of multianalyte assays [181]. An illustrative example is the WCB developed by Hui and colleagues, which integrated GFP and mCherry sensor systems to simultaneously monitor the bioavailability of cadmium and mercury in natural water environments [182]. This innovative system discriminates between mercury and cadmium pollution by measuring green and red fluorescence, respectively.

Microbial pigments offer another tool for biosensor construction, exploiting secondary metabolite gene pathways to produce colour changes indicative of the presence of target substances. This method simplifies observation, particularly in field applications, as colour changes are readily visible to the human eye. Common microbial pigments include pyocyanin, β-carotene, violacein, and indigoidine, each offering unique detection capabilities based on substrate availability and genetic expression [97,183,184,185].

A colourimetric output is also observed when the β-galactosidase is used as a reporter. It is an enzyme derived from *E. coli* that cleaves the β-galactose bonds of substrates such as X-galactopyranoside, realising a coloured product that can be quantified through spectroscopic methods [186,187]. Additionally, substrates generating luminescent or fluorescent products are also available, allowing versatile applications of this reporter [188,189,190]. Studies have found that β-galactosidase is relatively stable, but it requires an exogenous substrate. Owing to background noise derived from significant endogenous β-gal activity and the need for cellular lysis for the reporter measurement, only a few WCBs have been developed with *lacZ* as a reporter.

In the context of cold environments, the ice nucleation bacteria such as the psychrophilic *Pseudomonas syringae* and *Pseudomonas fluorescens* represent a source of an interesting class of proteins used as a reporter. Such bacteria, indeed, are known producers of ice nucleation proteins (INPs), which promote the formation of ice crystals at warmer temperatures than would occur spontaneously [191,192]. These proteins act as catalysts, effectively lowering the energy barrier required for ice nucleation to occur [193]. INPs play a crucial role in aiding the survival of bacteria in cold environments through various mechanisms [194]. Firstly, they prevent intracellular freezing by promoting the formation of ice crystals outside the cell, thus safeguarding cell membranes and proteins from damage that could lead to bacterial death. Additionally, INPs concentrate solutes around bacteria when water freezes, creating a protective environment that helps mitigate dehydration and cold-induced damage. Furthermore, these proteins initiate cryopreservation, a controlled freezing process used for preserving cells, by triggering the formation of ice crystals in a manner that facilitates bacterial survival at extremely low temperatures. Beyond their fundamental roles in nature, INPs hold significant implications for diverse applications, ranging from artificial snow production to cloud seeding [195,196]. Exploiting their unique properties, researchers have developed biotechnological systems in which INPs are used as a reporter. By transferring ice nucleation genes into chassis cells, these proteins serve as indicators of gene expression strength, allowing for the detection of target substances such as iron. In the study published by Loper and colleagues, INPs were utilized as reporters to investigate the effects of siderophores produced by rhizosphere microorganisms on iron availability in *P. putida* [197]. By employing a transcriptional fusion (*pvd-inaZ*) between an iron-regulated promoter (*pvd*, promoter of a pyoverdine production and uptake from *P. syringae*) and the ice nucleation reporter gene (*inaZ*), changes in iron levels were monitored. The expression of ina is measured by quantifying the ratio of ice nuclei to colony-forming units (CFU) using the droplet freezing assay and plating diluted culture. The differential expression of *ina* indicated that *P. putida* utilizes exogenous siderophores as a cheater bacterium since INA production was found to be inversely correlated with the concentration of and ferric–siderophore complexes in the culture media. While the INP reporting element ferric citrate can reduce the dependence on the bacterium phenotype properties, the detection process is complex and cannot achieve real-time dynamic monitoring.

Finally, in addition to the traditional optical reporting elements, recent innovations have explored electrochemical readouts to enhance biosensing capabilities [198]. They rely on microbe-electrode interactions where bacteria are embedded in a biofilm matrix connected to electrodes, which measure the current generated by a redox reaction in response to a specific analyte, such as heavy metals in water samples [199]. Despite these advancements highlighting the ongoing evolution and diversification of WCB technologies, further optimizations are needed to increase the specificity and sensitivity of these systems.

### 3.4. Host Features

To ensure real-time and accurate monitoring within marine waters, suitable chassis cells with specific features must be selected. *E. coli* is the most commonly chosen WCB organism for reasons linked to its ease of culture and transformation, availability of recombinant plasmids with diverse properties for exogenous gene expression, and straightforward genome editing strategies. However, the expression of recombinant constructs in different host cells may vary, necessitating the identification of the best-suited chassis cell to ensure optimal sensor functionality.

Several factors should be considered, primarily whether the microorganisms can respond to the specific analyte under stress conditions (Table 4). In the case of iron monitoring in polar seawater, selected bacteria must demonstrate effective iron uptake and storage systems to survive and grow in conditions of iron scarcity while also possessing detoxification mechanisms to survive in iron-rich marine regions near lands. Secondly, bacteria capable of growth at low temperatures are preferable. Examples of psychrophilic chassis cells exploited for monitoring heavy metals in marine waters include *P. putida* [110,200], *Pseudomonas fluorescens* [201], *Deinococcus radiodurans* [183], and *Shewanella oneidensis* [202]. However, none of these bacteria can grow at temperatures lower than 3–4 °C [203,204,205]. In a polar context, the availability of bacteria able to grow at subglacial temperatures in which recombinant expression systems have been developed can be very advantageous. In the literature, certain psychrophilic bacteria have been reported for their ability to produce recombinant proteins at low temperatures, such as *Pseudoalteromonas haloplanktis* TAC125. It is a γ-proteobacterium isolated from Antarctic coastal seawater able to grow in a wide range of temperatures comprised between −2.5 °C and 30 °C [206,207,208]. Its capability to produce recombinant proteins even at 0 °C, coupled with the development of recombinant systems for the production of fluorescent reporters, makes *P. haloplanktis* TAC125 a promising host for water quality monitoring applications in marine environments [209,210,211,212].

To effectively apply whole-cell biosensors (WCBs) in marine waters, it is crucial to use hosts able to thrive in nutrient-poor conditions and low temperatures, such as psychrophilic, psychrotolerant, and oligotrophic bacteria. Additionally, the in-field application of such systems requires strategies for a stable cell-on-chip integration that offers long-term storage and resilience to environmental factors. While planktonic cells are commonly used in WCB biosensor platforms, they often suffer from lower portability and storability due to the need for regrowth before each test. Therefore, alternative approaches, such as surface immobilization, where bacterial cells are immobilized on surfaces that support biofilm formation (e.g., glass, polystyrene, PVC), have demonstrated higher activity [215]. Surface immobilization can also offer better diffusion rates of analytes through thinner membranes, with cellulose-based filter membranes being a cost-effective and widely used option [77]. These membranes have been shown to preserve bacterial cells for extended periods, ensuring the viability of bioluminescent bacteria for monitoring water toxicity.

However, a fundamental aspect to consider in the development of WCBs for in situ measurements is the environmental risk associated with the potential release of genetically modified organisms into marine waters. To mitigate this risk, strategies to reduce the escape and horizontal gene transfer between recombinant and environmental bacteria are crucial. These strategies primarily rely on toxin–antitoxin systems (TAs), quorum sensing mechanisms, and bacterial encapsulation in eco-sustainable materials, ensuring that the WCBs remain effective and environmentally safe.

In the first case, TA systems are used to control bacterial proliferation by actuating a kill switch, which activates the toxin to disrupt an essential cellular process, such as DNA replication or protein synthesis, thereby inducing growth arrest under specific conditions [216]. In this way, the population growth of WCB can be controlled. Additionally, TA systems allow for stable plasmid maintenance, aiding in the distribution of plasmids to daughter cells and preserving genetic information across bacterial generations [217]. As for the quorum sensing-mediated control of bacterial growth, WCBs can be engineered so to produce signalling compounds known as autoinducers. When the bacterial population reaches a high cellular density, these autoinducers are secreted and then detected by the bacterial community, initiating specific responses such as growth arrest [218]. The encapsulation of the microbial cells within biocompatible alginate hydrogels also represents a strategy to limit uncontrolled bacterial proliferation in the marine environment. These hydrogels not only function as bacterial cell containment but also protect them from environmental stress, prolonging their shelf life [219].

Further, the WCB developed by Boyanapalli was specifically designed to integrate the Fe-responsive reporter cassette within the *desB* gene of *Synechococcus* sp. strain PCC 7002 [214]. The disruption of this gene generates a strain unable to grow at temperatures lower than 15 °C. As a result, the recombinant strain exhibits a phenotype unable to proliferate in the cold marine environment, addressing concerns regarding its potential spread.

Overall, the selection of appropriate chassis cells plays a crucial role in the effectiveness and reliability of biosensors for monitoring heavy metals and iron in marine waters, ensuring both environmental safety and accurate detection capabilities.

## 4. WCBs Application for Iron Monitoring in Seawater

The accurate detection of iron in marine waters is essential for understanding its role in oceanic biogeochemical processes and its impact on biodiversity preservation. While there are several examples of biosensors developed for iron detection in freshwater and wastewater systems [220,221,222,223,224], there remains a scarcity of such biosensors tailored specifically for marine environments (Table 5).

The first WCB for measuring iron bioavailability in seawater reported in the literature is based on a genetically modified heterotrophic bacterium, *P. putida* FeLux, able to produce bioluminescence in response to Fe limitation [34,225]. The FeLux bioreporter harnesses a chromosome-integrated *luxCDABE* cassette from *Aliivibrio fischeri* as a bioluminescent reporter gene whose expression is controlled by the *fepA–fes* promoter of *E. coli*, which acts as the sensing element since it is involved in the uptake of ferric enterobactin complex under the control of the Fur system. This means that when iron is limited, the promoter becomes active, leading to light production by the bacteria. Laboratory characterization revealed that the bioreporter responded sensitively to changes in Fe availability and demonstrated varying responses to different iron chelators, such as DFB, ferrichrome, DP, and RA [225]. After a first trial performed on freshwater samples collected from Lake Erie, which demonstrated that a substantial portion of bioavailable Fe is sequestred into particulate matter, the WCB was applied in oceanic waters. In field application, it was utilized in the FeCycle study, examining Fe fertilization in the sub-Antarctic Pacific, where changes in bioluminescence within an SF6-labeled patch of seawater were monitored to assess Fe availability changes for heterotrophic bacteria over time [34]. The results demonstrated that the developed WCB is so sensible as to respond to slight changes in iron levels in the marine environment (about 0.040 nM), providing data concerning the relationship between bioavailable and total dissolved iron. The significance of this study lay in suggesting differing responses of bacteria compared to phytoplankton to Fe fluctuations, thus improving the understanding of Fe cycling and microbial responses to its availability in marine environments. Limitations included the dependence on a single bacterial strain for the bioreporter response, with potential sensitivity variations across natural bacterial communities, necessitating further investigation to fully understand observed discrepancies between bacterial and phytoplankton responses. Overall, this research highlighted the potential of this kind of bioreporter for studying Fe dynamics in marine environments and emphasized the importance of considering different microbial groups when assessing Fe limitation.

The second example of WCB for iron monitoring in seawater was reported by Boyanapalli and coworkers, who developed a biosensor based on an engineered *Synechococcus* sp. strain PCC 7002 [214]. The bioreporter construction was performed by integrating the *isiAB* promoter with the *luxAB* gene. The sensing element is derived from the *isiAB* operon, belonging to the regulon controlled by Fur. It is constituted by the *isiA* gene, encoding a component of a light-harvesting complex known as Chl-binding protein, and the *isiB* gene, encoding flavodoxin protein involved in electron transfer reactions. When cyanobacteria experience iron deficiency, the *isiAB* operon is expressed, and the Chl-binding protein and the flavodoxin are produced. The *isiAB* promoter was, thus, fused to the *luxAB* reporter gene from *V. harveyi* to measure luminescence production in response to iron levels. After the biosystem characterization under a spectrum of growth conditions, the developed WCB was applied as complementary to chemical methods to measure Fe bioavailability on samples collected from the Baltic Sea and samples of the SERIES Fe fertilization experiment in the subarctic Pacific [214]. Although effective in defining iron limitation conditions and delineating dynamic iron cycling processes, these biosensors showed a limited sensitivity, needing optimization and validation before further application in Fe-deficient oceanic systems.

## 5. Summary and Outlook

Iron plays a fundamental role in oceanic biogeochemical processes, directly influencing the growth and productivity of marine organisms and, consequently, marine biodiversity. The use of whole-cell biosensors has shown promise in measuring iron in marine waters, offering crucial advantages over traditional methods, especially related to the measurement of bioavailable iron. These versatile biosensors enable the direct assessment of iron availability, offering insights into the repercussions of iron deficiency and enabling real-time monitoring of ecosystem responses to environmental fluctuations. However, it is important to acknowledge the limitations associated with the use of these tools, including biosensor sensitivity, the impact of environmental interferents, the need for accurate calibration, and the challenges in maintaining microbial viability and activity over the long term, both during storage and after exposure to samples.

Blanco-Ameijeiraset et al. comprehensively reviewed the limitations of utilizing *Synechococcus* sp. strain PCC 7002 as a WCB for iron detection, proposing strategies to increase sensitivity and extend its applicability to typical seawater low-Fe concentrations [226]. The study underscored challenges in achieving a monotonic dose–response curve, including issues related to cellular homeostasis, reporter enzyme, and sensor gene expression patterns. To overcome these challenges, the development of ‘light-off’ bioreporters with less energetically demanding reporter genes or using fluorescent proteins was suggested. New appropriate sensor genes for constructing the next generation of Fe-dependent cyanobacterial bioreporters suitable for open ocean systems were also proposed. Using differential transcriptomic analysis under varying Fe concentrations [227], they suggested genes expressing ATP-binding ACB transporters, such as *sufC*, for the development of “light-on” Fe WCBs. Otherwise, for “light-off” biosensors, they suggested genes involved in the electron transfer chain and Fe release from heme groups, such as *hoxE* and *ho*, respectively. Furthermore, the optimization of the bioluminescent response was considered, taking into account key factors such as exposure time, temperature, light intensity, and alternative luciferase substrates with longer half-times. Notably, the long-term acclimation to a mild iron limitation can be applied to enhance signal amplitude, thus improving accuracy in iron estimation.

An important consideration is that none of the strains used so far to construct WCBs for iron monitoring are psychrophilic. This review, therefore, looks forward to the development of Fe-sensing WCBs utilizing strains capable of growing in polar marine environments, such as the Southern Ocean. Polar marine bacteria represent a promising chassis component that can be practically used for real-time monitoring of seawater, unlike the described mesophilic ones, which are grown on sampled seawater treated at their optimal growth temperature (22–25 °C). Additionally, it is important to consider that another valid alternative is the use of photosynthetic organisms, such as phytoplankton, as they constitute the class of marine life whose growth is most significantly impacted by iron variation.

In the field of WCBs applied for heavy metal monitoring, numerous other strategies of optimizations have been applied to enhance their sensitivity, specificity, and robustness in diverse environmental conditions. Protein engineering based on rational design methods has been employed to modify transcription factors and promoter sequences, improving their response to specific metals [93,228]. Direct evolution approaches have also been utilized to select transcription factors with enhanced properties [93]. Genetic circuit design plays a crucial role, with the implementation of feedback loops or cascades amplifying signals and improving detection limits [229]. The integration of logic gates enhances detection specificity and selectivity, while multi-input circuits enable the simultaneous detection of multiple metals [229].

These optimization strategies pave the way for enhanced performance and the broader applicability of WCBs in iron detection in marine environments. By addressing key challenges such as sensitivity, specificity, and robustness, these strategies enable biosensors to provide more accurate and reliable measurements of iron concentrations in seawater. With improved biosensor technology, researchers and environmental scientists can better monitor iron availability in real time, allowing for timely interventions to mitigate iron deficiency and its ecological consequences. Thus, the continued advancement of biosensor optimization holds great promise for advancing our knowledge of marine biogeochemistry and supporting conservation efforts aimed at preserving marine biodiversity.

## Figures and Tables

**Table 3:** Reporter genes and their applications in whole-cell biosensors (WCBs).

Reporter Gene	Signal Type	Mechanism	Advantages	Disadvantages
Lux (Bacterial luciferase)	Bioluminescence	Emits blue-green light (490 nm) via oxidation of a long-chain aldehyde produced by luxCDE	No need for exogenous substrates	Heat-labile
Luc (Firefly luciferase)	Bioluminescence	Produces visible light using luciferin, ATP, oxygen, and magnesium ions	High sensitivity and signal stability	Requires external substrates
Aequorin	Bioluminescence	Emits blue light upon oxidation of coelenterazine in the presence of calcium ions	High sensitivity and signal stability	Requires external substrates
GFP (Green Fluorescent Protein)	Fluorescence	Fluoresces green upon exposure to specific wavelengths	Easy expression by a single gene	High background signal, slow maturation
RFP (Red Fluorescent Protein)	Fluorescence	Fluoresces red upon exposure to specific wavelengths	Allows multianalyte assays	Less brightness compared to GFP, more prone to photobleaching
CFP (Cyan Fluorescent Protein)	Fluorescence	Fluoresces cyan upon exposure to specific wavelengths	Allows multianalyte assays	Less brightness compared to GFP, spectral overlap with GFP, potential toxicity
YFP (Yellow Fluorescent Protein)	Fluorescence	Fluoresces yellow upon exposure to specific wavelengths	Allows multianalyte assays	Less brightness compared to GFP, sensitive to pH changes, prone to photobleaching
β-galactosidase (lacZ)	Colourimetric/Fluorescence	Cleaves X-gal to produce a coloured product; can also use luminescent/fluorescent substrates	Versatile applications	Requires exogenous substrates and cell lysis; endogenous β-gal activity can cause background noise
Ice Nucleation Proteins (INPs)	Physical/Visual	Promotes ice crystal formation at warmer temperatures	Suitable for cold environments	Complex detection process, not real-time
Microbial pigments	Colourimetric	Produces visible colour changes via secondary metabolite pathways	Easily observable in field applications	Dependent on substrate availability

**Table 4:** Main features of bacterial hosts used as WCB for marine water monitoring.

Host	Type of Organism	Growth Temperature Range	Analyte	References
*Escherichia coli*	Mesophilic	15–40 °C	Arsenic, Cobalt (II), Nickel (II), Mercury	[91,99,102,213]
*Shewanella oneidensis*	Mesophilic	5–30 °C	Nickel (II), Cadmium (II), Lead (II)	[202]
*Pseudomonas putida*	Mesophilic	8–35 °C	Arsenic, Copper, Mercury, Iron	[34,101,110,200]
*Deinococcus radiodurans*	Mesophilic	20–39 °C	Cadmium (II)	[183]
*Synechococcus* sp. strain PCC 7002	Mesophilic/Psychrotolerant	15–47.5 °C	Iron	[214]
*Pseudomonas fluorescens*	Psychrophilic	8–30 °C	Heavy metals	[201]

**Table 5:** Design and application of whole-cell biosensors for iron monitoring in polar waters.

Analyte	Sensing Element	Reporter Gene	Output	Chassis Cell	Field Application	Drawbacks	References
Bioavailable iron	*fepA–fes* from *E. coli*	*luxCDABE* from *V. fischeri*	bioluminescence	*P. putida* FeLux	Lake Erie; South West Pacific (FeCycle Fe fertilization study)	Sensitivity variations across natural bacterial communities	[34,225]
Bioavailable iron	*isiAB* promoter from *Synechocystis* sp. strain PCC 6803	*luxAB* from *V. harveyi*	bioluminescence	*Synechococcus* sp. strain PCC 7002	IOW 213, IOW 271, and Bocknis-Eck stations in the Baltic Sea; subarctic Pacific 50 km northeast of Ocean Station Papa (SERIES Fe fertilization study)	Low sensitivity, inadequate representation of picocyanobacteria diversity	[214]
